# Development of Gelatin-Based Renewable Packaging with *Melaleuca alternifolia* Essential Oil for Chicken Breast Preservation

**DOI:** 10.3390/polym17050646

**Published:** 2025-02-27

**Authors:** Rene Pereira de Lima, Daniela de Almeida Carrea, Vitor Augusto dos Santos Garcia, Cristina Tostes Filgueiras, Farayde Matta Fakhouri, José Ignacio Velasco

**Affiliations:** 1 Faculty of Engineering, Federal University of Grande Dourados, Dourados 79804-970, MS, Brazil; renepereira1714@gmail.com (R.P.d.L.); cristinafilgueiras@ufgd.edu.br (C.T.F.); 2 Poly2 Group, Department of Materials Science and Engineering, Universitat Politècnica de Catalunya (UPC Barcelona Tech), ESEIAAT, C. Colom 11, 08222 Terrassa, Spain; daniela.de.almeida.carrea@upc.edu; 3 Faculty of Agricultural Sciences, UNESP—São Paulo State University, José Barbosa de Barros, Botucatu 18610-034, SP, Brazil; vitor.as.garcia@unesp.br

**Keywords:** edible films, renewable packaging, *Melaleuca alternifolia*, chicken breast preservation, antimicrobial packaging, gelatin-based films

## Abstract

The aim of this study was to develop gelatin-based films incorporating Melaleuca alternifolia essential oil (MEO) and assess their application on refrigerated chicken breasts. The results showed that MEO exhibited antimicrobial activity against *Pseudomonas aeruginosa* and *Salmonella* sp., with inhibition zones of 17 mm and 9 mm, respectively. The minimum inhibitory concentration (MIC) was 10% for *P. aeruginosa* and 15% for *Salmonella* sp., demonstrating greater efficacy against *P. aeruginosa*. The antioxidant analysis using the ABTS method revealed activity of 1309 ± 18.0 μM Trolox/g, while the FRAP method resulted in 446 ± 5.78 μM FeSO_4_/g. The characterization of the oil by gas chromatography identified major compounds, including 2-carene, γ-terpinene, terpine-4-ol, and α-terpineol. Incorporating the oil into gelatin films resulted in structural changes, such as an increase in thickness (from 0.059 to 0.127 mm) and water vapor permeability. Furthermore, the addition of MEO conferred homogeneous properties to the films with no visible cracks. The incorporation of MEO into gelatin films has shown ABTS antioxidant activity, and FRAP results showed a significant increase with higher MEO concentrations. The packaged samples retained more mass than the control group, which lost about 90% of its weight during storage. Texture analysis revealed only an 8% variation in the Melaleuca-coated samples compared to 19.6% in the control group. These findings indicate that gelatin films containing *Melaleuca* essential oil effectively improve the shelf life of chicken breasts.

## 1. Introduction

Packaging made from renewable sources has been extensively studied due to significant environmental concerns surrounding the disposal of materials from non-renewable sources, such as petroleum. Edible packaging, in addition to its traditional composition—macromolecule, plasticizing agent, solvent, and pH adjuster, if necessary—can include some additives such as vitamins, bioactive compounds, and antimicrobials. These packaging types range from simple single-layer films to complex multi-layered structures [1,2,3,4,5,6,7,8].

Gelatin is widely produced for the national market and has been studied both in the production of films through the casting technique and in the preparation of film-forming solutions for application in different food products [9]. Gelatin does not only provide the foundational structure but also interacts with starch and the plasticizer, contributing to film flexibility [1]. The incorporation of essential oils enhances the mechanical, barrier, and antimicrobial properties of gelatin-based films [10].

Gelatin-stabilized CuO nanoparticles exhibited fungicidal activity and the potential for food packaging applications to extend shelf life [11]. The incorporation of green synthesized SnO2 nanoparticles from Laurus nobilis extract enhanced gelatin-based films by improving their hydrophobicity and antibacterial properties, leading to extended food shelf life [12]. Other studies showed an increase in the shelf life of fruits and vegetables coated with gelatin [1,10,13,14].

The antimicrobial activity of plant extracts is evaluated by determining the minimum amount of the substance required to inhibit microorganism growth; this value is known as the minimum inhibitory concentration (MIC) [15]. Determining the MIC value of plant extracts requires careful consideration of the toxicological, microbiological, and legal aspects pertinent to natural compounds or their combinations [16,17].

Species of the *Myrtaceae* family are distributed in tropical and subtropical regions [18]. The *Melaleuca* genus, native to Australia [19] and commonly known as the “tea tree”, mainly flowers in swampy areas near rivers [20]. It is widely used in the production of essential oil, which has been used in the cosmetic and pharmaceutical industries, primarily due to its therapeutic potential as a topical antifungal and antibacterial agent. The residue from its extraction has also been studied for the development of fibers as a reinforcing material in biofilms [19,21,22]. *Melaleuca alternifolia* essential oil (MEO) is also known for its antioxidant properties, which improve film deformation properties and provide strong antioxidant capacity, mechanical strength, and oxygen barrier [22,23,24].

Outbreaks of *Salmonella*-related foodborne are well known, involving various types of foods, particularly poultry products [25,26]. Studies in different countries have shown that 30 to 50% of frozen or refrigerated chicken carcasses are contaminated with *Salmonella* [27,28,29]. In Brazil, reports indicate the contamination of chickens and their products by *Salmonella* ranging from 9.15% to 86.7% [30,31]. As one of the world’s largest exporters of chicken meat, Brazil must ensure strict microbiological monitoring and control to ensure high food safety standards throughout the production process [32]. Research suggests that broiler chicken farms in Spain exhibit a greater diversity of Salmonella bacteriophages compared to layer farms, based on the most prevalent serovars [33].

In this context, the objective of this work was to develop gelatin-based films with the addition of *Melaleuca alternifolia* essential oil and to evaluate their application on refrigerated chicken breasts.

## 2. Materials and Methods

### 2.1. Materials

We collected Type A gelatin, Bloom 240, GAP 6 (Gelita^®^ do Brasil, Cotia, São Paulo, Brazil) and *Melaleuca alternifolia* oil (Nature’s Bond, Ronkonkoma, NY, USA). Chilled chicken breasts were purchased at the local market in Dourados—MS, Brazil. After acquiring the raw materials, they were immediately taken to the food technology laboratory at the School of Engineering, Federal University of Grande Dourados for processing. Additionally, the bacteria *Staphylococcus aureus* (ATCC 25923/29213), *Pseudomonas aeruginosa* (ATCC 27853), *Enterococcus faecalis* (ATCC 29212), *Salmonella* sp., *Escherichia coli* (ATCC 25922), and the culture media Mueller–Hinton agar (Himedia) and Mueller–Hinton broth (Himedia) were used.

### 2.2. Antimicrobial Activity

#### 2.2.1. Halo Test

The bacteria *Staphylococcus aureus* (ATCC 25923/29213), *Pseudomonas aeruginosa* (ATCC 27853), *Enterococcus faecalis* (ATCC 29212), *Salmonella* sp., and *Escherichia coli* (ATCC 25922) were activated in the Mueller–Hinton broth, and the inocula, consisting of bacterial culture in the logarithmic phase of growth were adjusted to a concentration equivalent to 0.5 on the McFarland scale, according to the methodology standardized by the National Committee for Clinical Laboratory Standards. After bacterial activation, the inocula were exposed to negative control, positive control, and sensitivity tests where each bacterium was inoculated onto Mueller–Hinton agar plates, followed by the addition of two 2 mm disks of films impregnated with *Melaleuca* oil. The plates were incubated at 36 °C for 24 h, and after this period, antibacterial activity was assessed by the presence or absence of inhibition halos around the disks. The disks that formed inhibition halos equal to or greater than 7 mm were considered active [34]. The size of the inhibition zone was determined by using a ruler.

For the negative control, a paper disk impregnated with only ethyl alcohol was used, and the positive control was based on the sensitivity of the bacteria to the antibiotics: Penicillin, Erythromycin, Gentamicin, and Vancomycin. All the tests were performed in triplicate.

#### 2.2.2. Minimum Inhibitory Concentration (MIC)

The minimum inhibitory concentration (MIC) of the oil was determined through serial dilution in the Mueller–Hinton broth, followed by the inoculation of each tube with the studied bacteria. Positive controls (culture medium + bacteria) and negative controls (culture medium + oil) were prepared, along with positive controls with Chloramphenicol. All the tests were performed in triplicate. Bacterial growth was assessed by measuring the optical density (OD) at 675 nm using a spectrophotometer. After visual analysis, a loopful from each tube was plated on the Mueller–Hinton agar and incubated at 36 °C for 24 h in an incubator. After this period, the plates were inspected for the observation of surface bacterial colony growth.

The MIC value was determined as the lowest oil concentration where no bacterial growth was observed [35].

### 2.3. Antioxidant Activity

#### 2.3.1. FRAP

The FRAP method (Ferric Reducing Antioxidant Power) was performed as described in [36]. In a dark environment, 25 mL of 0.3 M acetate buffer solution, 2.5 mL of 10 mM TPTZ solution (prepared with 40 mM HCl), and 2.5 mL of 20 mM ferric chloride solution were used. In triplicate, 90 µL of the sample was transferred to test tubes containing 270 µL of distilled water and 2.7 mL of freshly prepared FRAP reagent. The mixture was vortexed and kept in a water bath at 37 °C for 30 min. The reading was taken at 595 nm using a UV spectrophotometer (ZUZI MODEL 4201/50). The FRAP reagent was used as a blank to calibrate the spectrophotometer.

For antioxidant capacity quantification, a standard curve was constructed using aqueous solutions of ferrous sulfate. The antioxidant capacity was expressed in µM of ferrous sulfate per g of sample.

To verify if the films with MEO preserved their compounds, analyses were also performed on the films. Each film sample (~60 mg) was immersed in 20 mL of distilled water and stirred for 1 h at 40 °C. The mixture was then centrifuged at 5000 rpm for 5 min. A 90 µL aliquot of the supernatant was used for analysis [37].

#### 2.3.2. ABTS

Antioxidant capacity was also measured using ABTS (2,2′-azino-bis(3-ethylbenzothiazoline-6-sulfonic acid)). The ABTS radical was generated by reacting 5 mL of 7 mM ABTS stock solution with 88 μL of 140 mM potassium persulfate (K_2_S_2_O_8_) and then incubating it for 16 h in a dark environment. The ABTS solution was then diluted in ethanol to reach an absorbance (optical density) of 0.7 ± 0.02 at 734 ± 2 nm. In triplicate, 30 µL of the sample was added to test tubes containing 3 mL of the ABTS radical solution, vortexed, and left in the dark for 6 min. The reading was performed on a spectrophotometer at 734 nm (ZUZI MODEL 4201/50). Ethanol was used as a blank to calibrate the spectrophotometer.

The standard curve was adjusted using Trolox [6-hydroxy-2.5.7.8-tetramethylchroman-2-carboxylic acid] at concentrations ranging from 100 to 2000 µM. The results were calculated according to the equation adjusted by the standard curve and expressed in µM Trolox equivalents (TE) per g of sample [38]. Analyses were also performed on the films.

### 2.4. Oil Characterization

The *Melaleuca alternifolia* essential oil (MEO) was dissolved in hexane at a concentration of 100 µg/mL. The films were ground in a mortar and pestle before weighing. For the gelatin film samples, 100 mg was used, and the film compounds were extracted with 1 mL of hexane for 10 min using ultrasonic agitation.

The analyses were performed using a gas chromatograph equipped with a mass spectrometer detector (GCMS-QP2010 Ultra, Shimadzu, Kyoto, Japan). A DB-5 column (30 m length, 0.25 mm internal diameter, and 0.25 μm film thickness) was used, with helium (99.999% purity) as the carrier gas, at a flow rate of 1.0 mL/min and an injection volume of 1 μL (in split mode, 1:10). The initial oven temperature was set at 50 °C, with heating to 280 °C at 3 °C/min. The injector temperature was 220 °C, and the temperatures of the transfer line and the quadrupole detector were 280 °C. The MS scan parameters included electron impact ionization voltage at 70 V, a mass range from 50 to 550 Daltons, and a scan interval of 0.3 s. The retention index was calculated using a mixture of linear alkanes (C8–C30) as an external reference [39]. Compound identification was achieved by comparing the mass spectra of the samples with the spectra available in the NIST21 and WILEY229 libraries, as well as with data reported in the literature [39].

### 2.5. Film Preparation

The film-forming solution base was obtained by hydrating gelatin (10 g) in cold water for 1 h. The solution was then heated in a water bath (Nova Orgánica, São Paulo, Brazil) at 70 °C for ten minutes [40]. After the complete dissolution of the gelatin, the films were prepared using the casting technique. Gelatin-based formulations were as follows: (i) pure gelatin (control), (ii) gelatin with 5% Melaleuca oil, (iii) gelatin with 10% Melaleuca oil, (iv) gelatin with 15% Melaleuca oil, and (v) gelatin with 20% Melaleuca oil. The percentage of oil relative to the mass of the macromolecule was stirred on a heated plate (C-MAC HS 7, Ika, São Paulo, Brazil) for 4 min. After adding the oil, 25 mL of the film-forming solution was deposited onto Plexiglas^®^ plates with a diameter of 12 cm. For the control films, the same amount of film-forming solution was placed on the plate immediately after gelatin dissolution. All the films (with and without Melaleuca oil) were dried at 25 °C for 12 h and then stored in a desiccator at 53% relative humidity for 48 h prior to analysis.

### 2.6. Film Characterization

#### 2.6.1. Water Vapor Permeability

Water vapor permeability (WVP) was determined using the modified ASTM E-96 standard method (1980) [41]. The films were placed in acrylic permeation cells containing calcium chloride. Each cell was sealed with four screws and a rubber gasket shaped like the film to ensure that all the moisture permeation occurred solely through the film. The permeation cells were conditioned in desiccators maintained at 25 °C and 75% relative humidity. The water vapor transferred through the film was determined by measuring the increase in mass of the calcium chloride over 24 h intervals. The effect of air space, as described by McHugh and Krochta (1994) [42] between the area below the film and the surface of the calcium chloride, was not considered in the calculation of the water vapor transmission rate, as shown in Equation (1)

(1) $$\mathrm{WVP}=M\;\times \;\frac{e}{A\times \Delta P}$$

In which “*e*” is the mean film thickness (mm), “*A*” is the permeation area (m^2^), “Δ*P*” is the partial vapor pressure difference between two sides of films (kPa, at 25 °C), and “M” is the moisture absorption rate, calculated by the linear regression of weight gain and time, in steady state (g/h).

#### 2.6.2. Solubility

Film solubility was determined according to the method proposed by Gontard et al. (1994) [43]. The samples (circular, 20 mm in diameter) were dried, weighed, and immersed in a beaker containing 50 mL of distilled water. The system was maintained under slow agitation in a Dubnoff NT 232 digital water bath at 25 °C for 24 h. After 24 h, the samples were removed from the water and dried in an oven at 105 °C for 24 h to determine the final dry mass of the material that was not dissolved. The solubility (%S) was calculated using Equation (2):

(2) $$\%S=\frac{m_{1}-m_{2}}{m_{1}}\cdot 100$$

In which *m*_1_ is the initial dry mass of the films (g), and *m*_2_ is the final dry mass of on-solubilized films (g).

#### 2.6.3. Mechanical Proprieties

Tensile strength and elongation percentage at break were determined using a TA-XT2 texture analyzer (SMS, Surrey, UK), operated according to ASTM standard method D 882-83 [44], with an initial grip separation and probe speed of 50 mm and 1 mm/s, respectively. The films were cut into rectangles of 10 cm in length and 2.5 cm in width. The maximum force and extension at the point of rupture were determined.

### 2.7. Packaging of Chicken Breast

**Figure 1:**
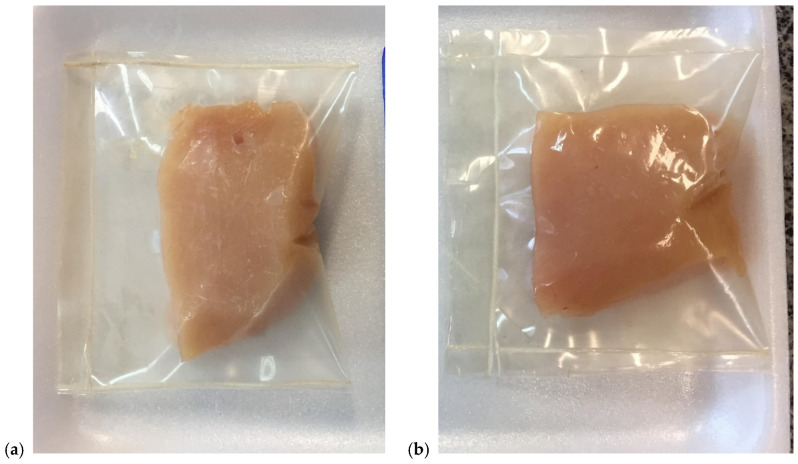
Chicken breast samples with control packaging (**a**) and packaging based on gelatin and tea tree oil (**b**).

Refrigerated chicken breast cuts were packaged with the films obtained in Section 2.3. The films were sealed using a pedal sealer (R.Baião, MG, Brazil) to create the final packaging (Figure 1). Samples without films were also prepared and stored under refrigeration at 5 °C for 10 days.

#### 2.7.1. Color Parameters

Color determination was performed using a colorimeter (Konica Minolta/CR-400/410) based on the parameters of lightness (L*), chroma a* (color variation parameter from green to red), and chroma b* (color variation parameter from blue to yellow). The readings were taken in triplicate both on the front and the back of the packaging.

Color difference (ΔE*) for each treatment was determined throughout storage relative to day 0 (zero). Δ*E* was also measured between treatments and the control sample on day 1 and day 10 according to Equation (1):

Δ*E* = [(L*_2_ − L*_1_)^2^ + (a*_2_ − a*_1_)^2^ + (b*_2_ − b*_1_)^2^]^1/2^ (3)

#### 2.7.2. pH

pH determination was carried out using a pH meter (PH—2000, Instrutherm), which was properly calibrated. Samples (3 g) were diluted in distilled water (100 mL). The mixture was stirred and homogenized with a glass rod until the particles were uniform for pH measurement. All the pH analyses were performed in triplicate.

#### 2.7.3. Texture Analysis

Texture analysis was conducted using a TA HDI texturometer (Stable Microsystems, Godalming, UK). The chicken breast samples were cut into cubes (2 × 2 cm) and stored in a refrigerator for a period of 9 days at a temperature of 5 °C ± 1. The analysis was performed following the direction of the muscle fibers, with a force expressed in kg, maintaining an initial distance of 10 mm, a cutting speed of 2 mm/s, and a return speed of 5 mm/s.

#### 2.7.4. Mass Loss

For the determination of mass loss, the fresh chicken samples and those packaged in films were initially weighed on day 0 using an Ohaus analytical balance (PA214CP). Subsequently, on days 1, 3, 5, 7, and 9, the mass loss was monitored, with the results expressed as a percentage.

#### 2.7.5. Antimicrobial

The antimicrobial activity of chicken breasts, both fresh and packaged in films, was evaluated against thermotolerant coliforms and *Salmonella* sp. using the MAPA method—Normative Instruction No. 62/AFNOR 3M 01/209/89C and ISO 6579:2002.

### 2.8. Statistical Analysis

All the analyses were performed in triplicate, and the results are presented as mean ± standard deviation. The comparison of means will be carried out using an analysis of variance (ANOVA), followed by Tukey’s test (*p* < 0.05).

## 3. Results

### 3.1. Antimicrobial Activity of Melaleuca alternifolia Essential Oil

Based on the results obtained, *Melaleuca alternifolia* essential oil (MEO) demonstrated antimicrobial activity against *P. aeruginosa* and *Salmonella* sp. (Table 1). MEO have well-documented antimicrobial properties against both Gram-positive and Gram-negative bacteria, fungi, and some viruses, as well as strong repellent activity against mosquitoes, fleas, and lice [45]. A previous study [46] showed that MEO and its components increased yeast cell permeability, enhanced plasma membrane fluidity, and inhibited extracellular medium acidification. Additionally, it was reported that the components of this oil exhibit multiple antifungal mechanisms of action. In this work, the inhibition zone for *P. aeruginosa* exposed to the oil was 17 mm, indicating high sensitivity to this antimicrobial (Table 1).

To ensure product safety and stability for the cosmetics industry, a previous study [47] tested the bacteriostatic and fungistatic activities of Copaiba, Rosemary, Melaleuca, Garlic, Andiroba, and propolis oils against *Staphylococcus aureus* (ATCC 6538), *Escherichia coli* (ATCC 8739), *Pseudomonas aeruginosa* (ATCC 9027), and *Candida albicans* (ATCC 10231). Among the analyzed samples, the best results were obtained with Melaleuca and Rosemary oils, which exhibited bacteriostatic and fungistatic activity against all four strains. In contrast, Andiroba, Copaiba, and Garlic oils showed no bacteriostatic or fungistatic activity against the tested microorganisms. In the case of propolis, inhibition zones were observed for *S. aureus* (Gram-positive) and *E. coli* (Gram-negative), but no inhibition was detected for *P. aeruginosa* (Gram-negative).

Regarding *Salmonella*, an inhibition zone of 9 mm was observed in response to MEO (Table 1). While [48] suggests that inhibition zones with diameters below 12 mm may not indicate significant antibacterial activity for plant extracts, the observed antimicrobial activity of MEO against *P. aeruginosa and Salmonella* sp., prompted a determination of the minimum inhibitory concentration (MIC) of the oil.

The results showed that the MIC of MEO was 10% for *P. aeruginosa* and 15% for *Salmonella* sp. (Table 2). This demonstrates the higher antimicrobial activity of the oil against *Pseudomonas* compared to *Salmonella*. The effective inhibition of *P. aeruginosa* is a promising result, as this microorganism is a member of the most important group of spoilage bacteria in refrigerated fresh foods [49], particularly meat, where it can grow at refrigeration temperatures of 2 to 5 °C. The growth of *Pseudomonas* in meat is associated with intense proteolytic activity, leading to a marked increase in NPN (non-protein nitrogen) levels, especially in the form of peptides and ammonia [50].

A previous study [51] investigated the activity of MEO incorporated into gel and lotion formulations at concentrations of 10%, 15%, 20%, and 25%, which were tested against strains of the yeast *Candida albicans*, *C. tropicalis*, and *C. glabrata* using the Pour-Plate seeding technique. The results showed the formation of inhibition halos in the tested formulations for the strains of *C. albicans* and *C. tropicalis*, thereby demonstrating its antifungal action in vitro.

The MIC value of MEO against strains of *C. albicans*, *C. krusei*, and *C. tropicalis* was determined by several studies, which identified inhibitory action at concentrations ranging from 0.06% to 0.5% [46,52,53]. The minimum fungicidal concentration (MFC) has also been reported within a similar range from 0.125% to 0.5%. These findings indicate that the MEO exhibits antifungal activity at concentrations below 1%, thereby highlighting its significant antimicrobial potential.

### 3.2. Antioxidant Activity of Melaleuca alternifolia Essential Oil

The result showed an antioxidant activity of 1309 ± 18.0 μM Trolox/g, corresponding to a radical inhibition of 57.87% determined using the ABTS method (Table 3).

An antioxidant analysis using the ABTS method on MEO acquired from the brand Aura Cacia (Norway, IA, USA) obtained a result of 7.6 ± 1.0 mmol/L in the fresh oil [54]. These values are lower than those found in the present study, where the conversion resulted in 13.48 ± 0.18 mmol/L.

The results for FRAP in MEO are higher than those reported for Lemon, Cinnamon, Lemon balm, Cedar, Mandarin, and Rosemary essential oils, which measured 11.250 ± 900, 12.549 ± 753, 7.276 ± 291, 5.996 ± 239, and 4.798 ± 288 μM Fe/100 g EO, respectively. However, they are lower than the values for Clove and Thyme, which were 62.273 ± 3000 and 60.840 ± 1.825 μM Fe/100 g EO, respectively [55].

The antioxidant effect exhibited by essential oils is also significant for food preservation, particularly those rich in lipids [56].

### 3.3. Oil Characterization

**Figure 2:**
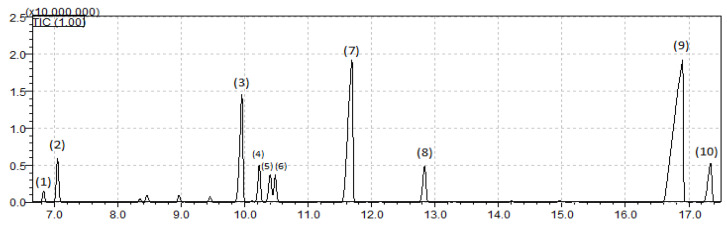
Mass spectra. (1) α-thujene; (2) α- pinene; (3) 2-carene; (4) α-terpinene; (5) limoneno; (6) 1,8-cineole; (7) y-terpinene; (8) mentha-3,8-diene; (9) terpine-4-ol; (10) α-terpineol.

The main compounds identified by gas chromatography were α-pinene, 2-carene, α-terpinene, γ-terpinene, mentha-3,8-diene, terpine-4-ol, and α-terpineol, found in the MEO and in films with different concentrations of essential oil. However, it was observed that the highest concentrations of these compounds were found in the films produced with the addition of 20% essential oil, 2-carene (23.4%), γ-terpinene (51.2%), terpine-4-ol (78.9%), and α-terpineol (9.2%). Additionally, compounds such as aromadandrene, spathulenol, β-pinene, and myrcene were only observed in the essential oil and in the film with the highest concentration of essential oil (Table 4). The mass spectra of the identified compounds are shown in Figure 2.

### 3.4. Characterization of Packaging Films

#### 3.4.1. Visual Assessment

The films obtained were easily manageable and removable from the plate, regarded as homogeneous, with no brittle areas or visible cracks. The addition of MEO resulted in a slight color change compared to the control sample.

#### 3.4.2. Water Vapor Permeability (WVP) and Solubility

Table 5 presents the characterization of films incorporating MEO, focusing on thickness, WVP, and solubility. The thickness of the films ranged from 0.059 to 0.127 mm. A significant difference in thickness was observed between the control films and those containing MEO, suggesting that the incorporation of higher concentrations of oil may influence film formation. This observation aligns with the findings of Wu et al. (2017) [57], who reported that the addition of essential oils to gelatin films can reduce the interaction between protein molecules, leading to less compact textures with a porous structure and, consequently, increased film thickness.

Regarding water vapor permeability (WVP), all the films containing MEO exhibited higher WVP values compared to the control films (pure gelatin). However, irrespective of the concentration of oil added, no significant differences were observed among the films, with values approximately equal to 3 (g.mm/h.m^2^.kPa). Atares et al. (2016) [58] highlighted that the impact of lipid addition on the microstructure of the film is a critical factor in determining water barrier efficiency. The incorporation of essential oil into gelatin films results in a less compact structure compared to pure gelatin films, potentially facilitating increased pathways for water vapor transfer, thus contributing to a rise in WVP.

The control gelatin films exhibited a solubility of 30.98%, significantly lower than those with added essential oil, which demonstrated higher solubility (~44%). This could be attributed to the hydrophobic nature of the essential oil, which may hinder water vapor penetration through the film, whilst the hydrophilic properties of gelatin can enhance moisture retention, thereby increasing solubility.

#### 3.4.3. Mechanical Proprieties

**Figure 3:**
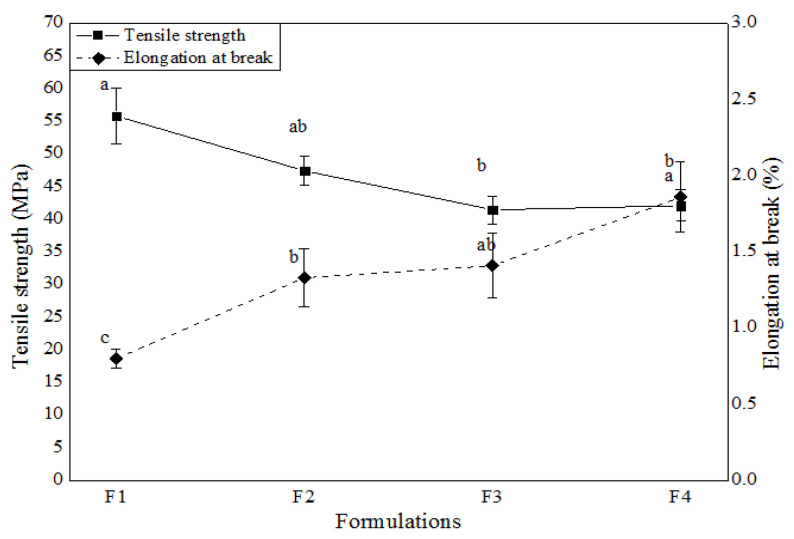
Mechanical properties (tensile strength and elongation) of gelatin-based films with different concentrations of MEO: F1 (5%), F2 (10%), F3 (15%), and F4 (20%). different lowercase letters in the same row indicate significant differences (*p* < 0.05) for different formulations.

Mechanical properties are shown in Figure 3. The addition of essential oil causes a decrease in tensile strength and an increase in film elongation. 

#### 3.4.4. Antioxidant Activity

The antioxidant activity of MEO films was evaluated using FRAP and ABTS assays (Table 6). The FRAP results showed a significant increase (*p* < 0.05) with higher MEO concentrations. The control sample exhibited the lowest activity (6.23 ± 4.28 μM FeSO_4_/g), while the 20% MEO film reached the highest value (136.26 ± 1.38 μM FeSO_4_/g). Tukey’s test confirmed significant differences between all the formulations.

The ABTS assay demonstrated a progressive increase in radical scavenging activity with increasing MEO concentrations. While films with 5% and 10% MEO showed no significant difference (*p* > 0.05), higher concentrations (15% and 20%) exhibited markedly improved antioxidant capacity. The 20% MEO film demonstrated the highest ABTS inhibition (54.39 ± 2.97%).

These results confirm the successful incorporation of MEO into the polymer matrix, leading to a proportional enhancement of antioxidant properties.

The incorporation of bioactive compounds into gelatin–sodium alginate films has shown an ABTS antioxidant activity of 53.36 mg (TE/g film) [59], which is lower than the result obtained for 20% MEO films (291.37 *±* 7.4 TE/g film*)*. Kilinc et al. (2021) [60] found a higher ABTS radical inhibition with gelatin film incorporated with 4% *Origanum onites* L. essential oil (61.57%). The result of FRAP was lower when compared with gelatin-lignin film (180.20 ± 5.71 μM Fe^2+^/g film) [61].

Studies showed that the incorporation of active compounds into gelatin films preserved their properties, such as gelatin films with red propolis extract (25%) exhibited strong antioxidant effects, improving the overall stability and quality of the gelatin films [62].

### 3.5. Application of Packaging on Chicken Breast

#### 3.5.1. Color Parameters

The results of the color analysis are shown in Table 6. While the control (without packaging) sample varied by approximately 13% throughout the experiment, the samples with packaging showed variations of 4% and 6% for the control packaging (FC) and in the packaging with MEO (FM), respectively, indicating that the packaging contributed to a reduced color variation during the storage period.

A slight color variation (Table 7) was observed between the control samples and the packaged samples on both the first and last days of storage.

#### 3.5.2. pH

Table 8 shows that the pH values of the chicken breasts stored without gelatin packaging did not present a significant difference between the first and last day of storage. In contrast, the samples stored in edible packaging, both with and without the addition of MEO, exhibited a reduction in pH after 9 days of storage at 5 °C, showing a significant difference compared to the first day. According to Roça (2006) [63], the average pH of chicken meat is 5.87, indicating that the addition of gelatin-based edible packaging, with or without MEO, did not significantly influence the pH of chicken breasts, remaining close to the control. After slaughter, the pH of chicken meat decreases due to acid formation, with the final pH of breast meat expected to be between 5.7 and 5.9.

Monitoring pH during the storage period is crucial for assessing meat quality, as an increase in pH, often caused by substances formed by the metabolism of spoilage bacteria, can lead to meat deterioration.

After the fifth day of storage, a significant difference in pH was observed for the chicken breast packaged with essential oil compared to the control and FC samples. This may indicate a possible migration of the essential oil into the chicken breasts, contributing to a reduction in pH, which could result in greater stability for this sample.

#### 3.5.3. Texture Analysis

Table 9 presents the texture analysis of the chicken breasts with and without the addition of edible packaging. The packaged samples were more effective in preserving texture compared to the control sample. It can be observed that the FM sample with the addition of Melaleuca oil was more stable regarding texture analysis after the storage period, showing a maximum variation of 8%, while the control sample exhibited a texture variation of 19.6%.

#### 3.5.4. Mass Loss

**Figure 4:**
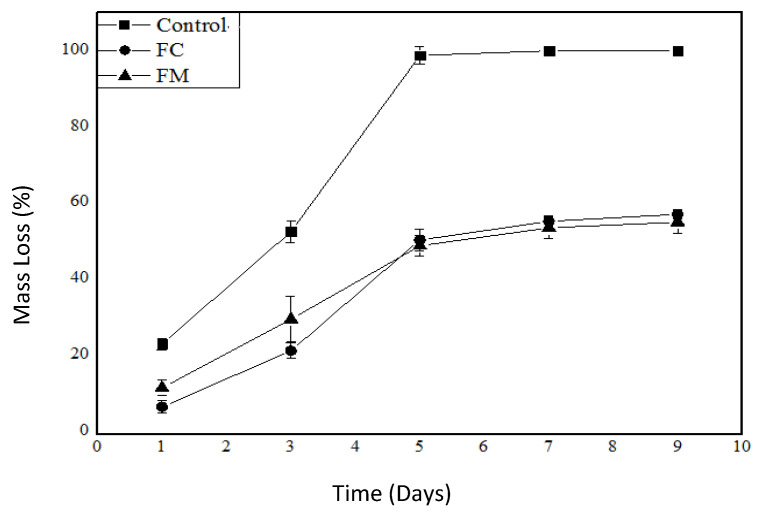
Mass loss in the chicken breasts without the packaging (control), in the control packaging (FC), and in the packaging with MEO (FM) as a function of storage days.

Analyzing the mass loss graph (Figure 4), we can observe a significant difference between the control and the other samples; clearly, the control exhibited an approximate mass loss of 90% during the studied period. The FC and FM treatments showed a maximum mass loss of about 50% during the study period, a percentage that the control sample reached by the third day of storage. These data emphasize that the packaging was effective when this parameter was evaluated.

According to the MAPA normative instruction number 8 from 2010, the moisture content of boneless, skinless chicken breasts for commercialization should have a lower limit of 73.3% and an upper limit of 75.8%. Thus, the reduction in mass loss achieved by the developed packaging compared to the control can contribute to an extended shelf life of the product.

#### 3.5.5. Antimicrobial Activity

The total count of thermotolerant coliforms at 45 °C remained constant among the treatments until the ninth day of storage (Table 10). According to Resolution RDC No. 12 from National Health Surveillance Agency of Brazil 2001, for fresh poultry, whether refrigerated or frozen, the maximum tolerance for total thermotolerant coliforms at 45 °C is 10^4^ CFU/g, and the absence of *Salmonella* sp. in 25 g of the sample is required. Therefore, the fresh chicken breasts (time 0), packaged with gelatin and with MEO (day 9), demonstrated compliance with microbiological standards, which may contribute to a longer commercialization period for the product. The active film containing MEO provided balance in the microbiological count of the packaged meats, confirmed by the pH values (Table 8), while also maintaining the desired color of the meat for a longer period (Table 7).

## 4. Conclusions

Melaleuca alternifolia essential oil (MEO) demonstrated antimicrobial activity against *P. aeruginosa* and *Salmonella* sp. with MIC values of 10% and 15%, respectively. *Salmonella* sp. is particularly concerning due to its impact on poultry health. The results also showed antioxidant activity of 1309 ± 18.0 μM Trolox/g, corresponding to a radical inhibition of 57.87% as determined by the ABTS method. These findings suggest that MEO can be effectively used as a natural antimicrobial and antioxidant agent in food packaging applications.

The addition of MEO increased elongation at break and water vapor permeability while decreasing the tensile strength and solubility of the gelatin films. Also, the incorporation of MEO into gelatin films has shown an ABTS antioxidant activity and FRAP results showed a significant increase with higher MEO concentrations.

The active film containing MEO helped maintain a balanced microbiological count in the packaged meats, as confirmed by the pH values, while also preserving the desired color of the meat for a longer period. These results suggest that MEO-containing films could serve as either a primary or supplemental packaging solution for better food preservation.

In conclusion, films containing MEO offer a promising approach for extending the shelf life of chicken breasts within rapid commercialization systems, combining antimicrobial and antioxidant protection with desirable characteristics such as biodegradability and sustainability.

## Figures and Tables

**Table 1:** Inhibition zone diameters (mm) measured for the bacteria *Escherichia coli*, *Staphylococcus aureus*, *Pseudomonas aeruginosa*, and *Salmonella* sp. in response to MEO.

Bacteria	Inhibition Zone Diameter (mm)
*Escherichia coli*	Ø
*Staphylococcus aureus*	Ø
*Pseudomonas aeruginosa*	17
*Salmonella* sp.	9

The symbol “Ø” represents no inhibition detected.

**Table 2:** Minimum inhibitory concentration (MIC) of MEO against *Pseudomonas aeruginosa* and *Salmonella* sp.

Bacteria	Minimum Inhibitory Concentration (%)
*Pseudomonas aeruginosa*	10
*Salmonella* sp.	15

**Table 3:** Antioxidant activity of MEO.

	FRAP (μM FeSO_4_/g)	ABTS (μM Trolox/g)
Melaleuca EO	446 ± 5.78	1309 ± 18.0

**Table 4:** Monoterpene and sesquiterpene chemical characterization by gas chromatography of gelatin films (GFs).

Compounds (mg/g of GF)	Concentration			
	5%	10%	15%	20%
α-Thujene	---	---	1.00	1.50
α-Pineno	1.50	3.10	4.70	6.60
Canrphene	---	---	---	---
Sabinene	---	---	---	1.00
β-Pinene	---	---	---	1.00
Myrcene	---	---	---	1.00
2-Carene	6.40	11.50	18.20	23.40
α-Terpinene	1.50	3.10	4.50	6.40
Limoneno	1.40	2.70	4.10	5.60
1,8-Cineole	1.20	2.40	3.60	4.90
β-Ocimene	---	---	---	---
γ-Terpinene	11.00	21.70	33.10	51.20
Mentha-3,8-diene	1.60	3.20	4.90	6.80
α-Terpinene	---	1.10	1.50	2.10
Torreyol1	---	1.20	1.50	2.30
Terpine-4-ol	20.10	39.50	59.70	78.90
α-terpineol	2.30	4.70	6.10	9.20
Myrrenol	---	---	---	---
Copene	---	---	---	---
Cyperene	---	---	---	---
Caryophyllene	---	1.50	2.30	3.10
Aromadandrene	---	---	---	1.00
Cunracrene		1.10	1.40	2.50
α-Selinene	---	---	1.00	1.40
Cubebol	---	---	---	---
Spathulenol	---	---	---	1.00
Globulol	---	---	---	---
Guaiol	---	---	---	---
Cedren9-one	---	---	---	---

**Table 5:** Characterization of gelatin film with different concentrations of MEO.

Sample	Thickness (mm)	*WVP* (g.mm/h.m^2^.kPa)	Solubility (%)
Control	0.059 ± 0.003 ^b^	1.72 ± 0.10 ^b^	30.98 ± 2.98 ^b^
5%	0.107 ± 0.016 ^a^	2.90 ± 0.52 ^a^	44.15 ± 3.41 ^a^
10%	0.114 ± 0.022 ^a^	2.96 ± 0.32 ^a^	44.46 ± 2.67 ^a^
15%	0.127 ± 0.033 ^a^	3.33 ± 0.77 ^a^	44.61 ± 0.51 ^a^
20%	0.126 ± 0.004 ^a^	3.32 ± 0.27 ^a^	43.34 ± 5.57 ^a^

Different lowercase letters in the same row indicate significant differences (*p* < 0.05) for different formulations.

**Table 6:** Antioxidant activity of the films.

Sample	FRAP (μM FeSO_4_/g)	ABTS (%)
Control	6.23 ± 4.28 ^a^	-
5%	23.95 ± 0.75 ^b^	2.42 ± 0.79 ^a^
10%	50.17 ± 7.10 ^c^	3.92 ± 0.60 ^a^
15%	78.43 ± 4.91 ^d^	26.39 ± 1.64 ^b^
20%	136.26 ± 1.38 ^e^	54.39 ± 2.97 ^c^

Different lowercase letters in the same row indicate significant differences (*p* < 0.05) for different formulations.

**Table 7:** Color analysis of fresh chicken breasts stored at 5 °C for 9 days.

Time (Days)	Time (Days)	L*	a*	b*	ΔE*	ΔE**
0	Control FC FM	59.79 ± 2.44 55.41 ± 1.05 58.01 ± 1.74	4.45 ± 1.43 4.21 ± 1.26 2.97 ± 0.74	6.70 ± 1.71 9.27 ± 1.24 9.69 ± 2.15	12.83 4.22 6.37	- 5.08 4.00
9	Control FC FM	49.68 ± 1.97 55.25 ± 2.95 53.05 ± 1.96	9.87 ± 2.63 6.65 ± 1.34 6.97 ± 1.09	12.45 ± 2.27 12.71 ± 1.43 12.94 ± 1.79	- - -	6.44 4.47

ΔE*—difference in color between the treatments throughout storage. ΔE**—difference in color between the chicken breasts without packaging (control) and control packaging (FC) and between the chicken without packaging (control) and in packaging with MEO (FM) on the first and last day of storage.

**Table 8:** pH analysis of fresh chicken breasts stored at 5 °C for 9 days.

Time (Days)	Control ^1^	FC ^2^	FM ^3^
0	5.81 ± 0.06 ^Ca^	5.81 ± 0.06 ^Ca^	5.81 ± 0.06 ^Ca^
1	6.02 ± 0.01 ^Aa^	5.86 ± 0.02 ^BCb^	5.82 ± 0.03 ^BCc^
3	6.01 ± 0.09 ^Aba^	5.96 ± 0.13 ^ABa^	5.88 ± 0.08 ^ABa^
5	5.94 ± 0.05 ^Bab^	5.97 ± 0.04 ^Aa^	5.88 ± 0.06 ^ABb^
7	5.97 ± 0.06 ^Aba^	5.98 ± 0.06 ^Aa^	5.90 ± 0.02 ^Ab^
9	5.84 ± 0.06 ^Ca^	5.70 ± 0.11 ^Db^	5.59 ± 0.01 ^Dc^

^1^ without packaging; ^2^ control film; ^3^ MEO film. Uppercase letters in the same column indicate significant differences (*p* < 0.05) for the same formulation, and different lowercase letters in the same row indicate significant differences (*p* < 0.05) for different formulations.

**Table 9:** Texture analysis of fresh chicken breasts stored at 5 °C for 9 days.

Time (Days)	Sample	Control	20%
0	0.402 ± 0.02 ^Ca^	0.402 ± 0.02 ^Ca^	0.402 ± 0.01 ^Ca^
1	0.414 ± 0.02 ^Ca^	0.412 ± 0.01 ^Ca^	0.413 ± 0.01 ^Ba^
3	0.410 ± 0.01 ^Cb^	0.412 ± 0.01 ^Ca^	0.412 ± 0.01 ^Ba^
5	0.472 ± 0.02 ^ABa^	0.417 ± 0.03 ^ABb^	0.435 ± 0.01 ^Ab^
7	0.482 ± 0.01 ^Aa^	0.418 ± 0.01 ^Ab^	0.428 ± 0.01 ^Ab^
9	0.462 ± 0.07 ^Ba^	0.423 ± 0.01 ^Bb^	0.424 ± 0.01 ^ABb^

Uppercase letters in the same column indicate significant differences (*p* < 0.05) for the same formulation, while different lowercase letters in the same row indicate significant differences (*p* < 0.05) for different formulations.

**Table 10:** Microbiological assessment of the packaged and unwrapped chicken breasts against coliforms at 45 °C and *Salmonella* sp. on the first and last day of storage.

	Time (Days)			
	0		9	
	Coliformes, 45°C (UFC/g)	*Salmonella* sp.	Coliformes, 45 °C (UFC/g)	*Salmonella* sp.
Control ^1^	<1.0 × 10^2^	Ø	<1.0 × 10^2^	Ø
FC ^2^	<1.0 × 10^2^	Ø	<1.0 × 10^2^	Ø
FM ^3^	<1.0 × 10^2^	Ø	<1.0 × 10^2^	Ø

The symbol Ø represents no inhibition detected. ^1^ without packaging; ^2^ control film; ^3^ MEO film.

## Data Availability

Data are contained within the article. The original contributions presented in this study are included in the article. Further inquiries can be directed to the corresponding authors.
